# Network-Guided Drug Repurposing for Hantaan Virus Infection in Endothelial Cells: Pathway Analysis, Target Identification, and Mechanistic ODE Simulations

**DOI:** 10.3390/v18101100

**Published:** 2026-10-04

**Authors:** Pietro Hiram Guzzi, Francesco Branda, Pierangelo Veltri, Federico Manuel Giorgi

**Affiliations:** 1 Department of Surgical and Medical Sciences, University ‘Magna Graecia’ of Catanzaro, 88100 Catanzaro, Italy; hguzzi@unicz.it; 2 Unit of Medical Statistics and Molecular Epidemiology, University Campus Bio-Medico of Rome, 00128 Rome, Italy; f.branda@unicampus.it; 3 Department of Computer Engineering, Modeling, Electronics and Systems Engineering, University of Calabria, 88100 Rende, Italy; pierangelo.veltri@unical.it; 4 Department of Pharmacy and Biotechnology, University of Bologna, 40126 Bologna, Italy

**Keywords:** hantavirus, drug repurposing, interferon signalling, network medicine, ISG15, STAT1, CXCL10, RIG-I, JAK inhibitors, ODE modelling

## Abstract

Hantaviruses cause haemorrhagic fever with renal syndrome and hantavirus cardiopulmonary syndrome, which are together the cause of more than 200,000 human cases every year. However, no specific antiviral therapy is approved. Here, we develop a three-stage computational framework for systematic drug repurposing. Transcriptome-wide analysis of hantavirus-infected human endothelial cells showed a response dominated by interferon pathways. A STRING protein–protein interaction network and a composite network centrality score identified 15 hub genes, including ISG15, IRF1, CXCL10, STAT1, and DDX58. Druggability and literature analysis of these hubs provided 12 repurposing candidates across four priority tiers. An ordinary differential equation (ODE) model of type I interferon signalling then evaluated eight drug perturbations. Early IFN-*β* achieved the fastest viral clearance (3.5 days) with reduced CXCL10 output. Early JAK inhibition delayed clearance (4.9 days) and increased IRF1-driven CXCL10. Our simulation showed that IFN-*β* combined with an anti-CXCL10 monoclonal antibody produced the best balance between antiviral effect and CXCL10 output. A structural sensitivity analysis showed the antiviral predictions to hold under an alternative network topology, while the chemokine predictions depended on it. This computational pipeline prioritises candidate therapies and phase-specific combinations for preclinical hantavirus studies.

## 1. Introduction

Hantaviruses (family *Hantaviridae*, order *Bunyavirales*) are enveloped, single-stranded RNA viruses with a negative-sense tripartite genome encoding the nucleocapsid protein (N), glycoproteins (Gn/Gc), and the RNA-dependent RNA polymerase (RdRp) [1]. These viruses are transmitted primarily through inhalation of aerosolized droplets from infected rodent reservoirs and cause two potentially lethal syndromes: haemorrhagic fever with renal syndrome (HFRS), prevalent in Europe and Asia and caused principally by Hantaan (HTNV), Seoul, Puumala, and Dobrava viruses; and hantavirus cardiopulmonary syndrome (HCPS), an endemic disease in North and South America caused by Sin Nombre, Andes, and related viruses [2]. Collectively, these pathologies account for more than 200,000 human cases every year, with fatality rates ranging from 1% (HFRS) to 40% (HCPS) [1]. Despite this evident burden for public health, clinical strategies for hantavirus cases remains largely supportive, and no specific antiviral therapy has been approved yet [3].

In *Homo sapiens*, the early response against hantavirus infection is carried out by the host innate immune system, which triggers strong activation of the type I interferon (IFN-I) pathway through cytosolic receptors, particularly RIG-I (encoded by the gene DDX58) and MDA5 (encoded by IFIH1), which detect viral RNA. These receptors trigger downstream signalling cascades that culminate in IFN-beta secretion and the transcription of hundreds of interferon-stimulated genes (ISGs), such as CXCL10, ISG15, GBP1, OAS1, IFIT1, and MX1 [4,5]. ISGs hinder viral replication via molecular defences such as particle trapping, translation inhibition, RNase L activation, and membrane disruption [6]. However, both IRF1 and STAT1 transcription factors can induce a prolonged and deleterious activation of the ISG programme, with CXCL10 in particular recruiting T and natural killer (NK) lymphocytes to the infected endothelium, and ultimately leading to sustained inflammation, vascular leakage, cytotoxicity, and tissue damage [2,3,7,8]. This dual effect of the IFN-I pathway creates a therapeutic dilemma: enhancing it risks a CXCL10-associated immunopathology, while reducing it too much suppresses inflammation and leaves free rein to the virus.

Via the identification of the most influential nodes in host–pathogen interaction networks, network medicine offers a rational strategy to navigate this dilemma, translating abstract network hubs into prioritised therapeutic targets [9,10], an approach already successfully applied to SARS-CoV-2 and other viral infections [9].

Timing the intervention is as important as *what* to target. For example, JAK inhibitors have been shown to improve viral propagation when administered during viral replication [11], highlighting the need for phase-specific treatments. Ordinary differential equation (ODE) models of signalling cascades allow one to model such time-dependent effects in silico before committing to animal experimentation or clinical trials [12,13].

Here, we present a three-stage computational strategy to repurpose drugs as candidate effectors against hantavirus infection. The steps are: (1) transcriptomic characterisation of the HTNV host response; (2) PPI network analysis and druggability assessment to identify and prioritise 12 repurposing candidates; (3) ODE-based molecular what-if simulations of eight drug perturbation scenarios, providing molecular insight into the timing and combination strategies that are most likely to achieve both antiviral efficacy and control of the downstream output of CXCL10. Our proposed framework identifies specific candidates via integration of all steps and also, traditionally, through the steps of the pipeline itself: transcriptomics analysis, PPI network analysis, network hub prioritisation, druggability assessments, and investigation of drug-perturbation data. Our work illustrates how heterogeneous biological and pharmacological knowledge can be merged to create experimentally testable hypotheses.

## 2. Materials and Methods

### 2.1. Transcriptomic Data and Differential Expression Analysis

**Figure 1:**
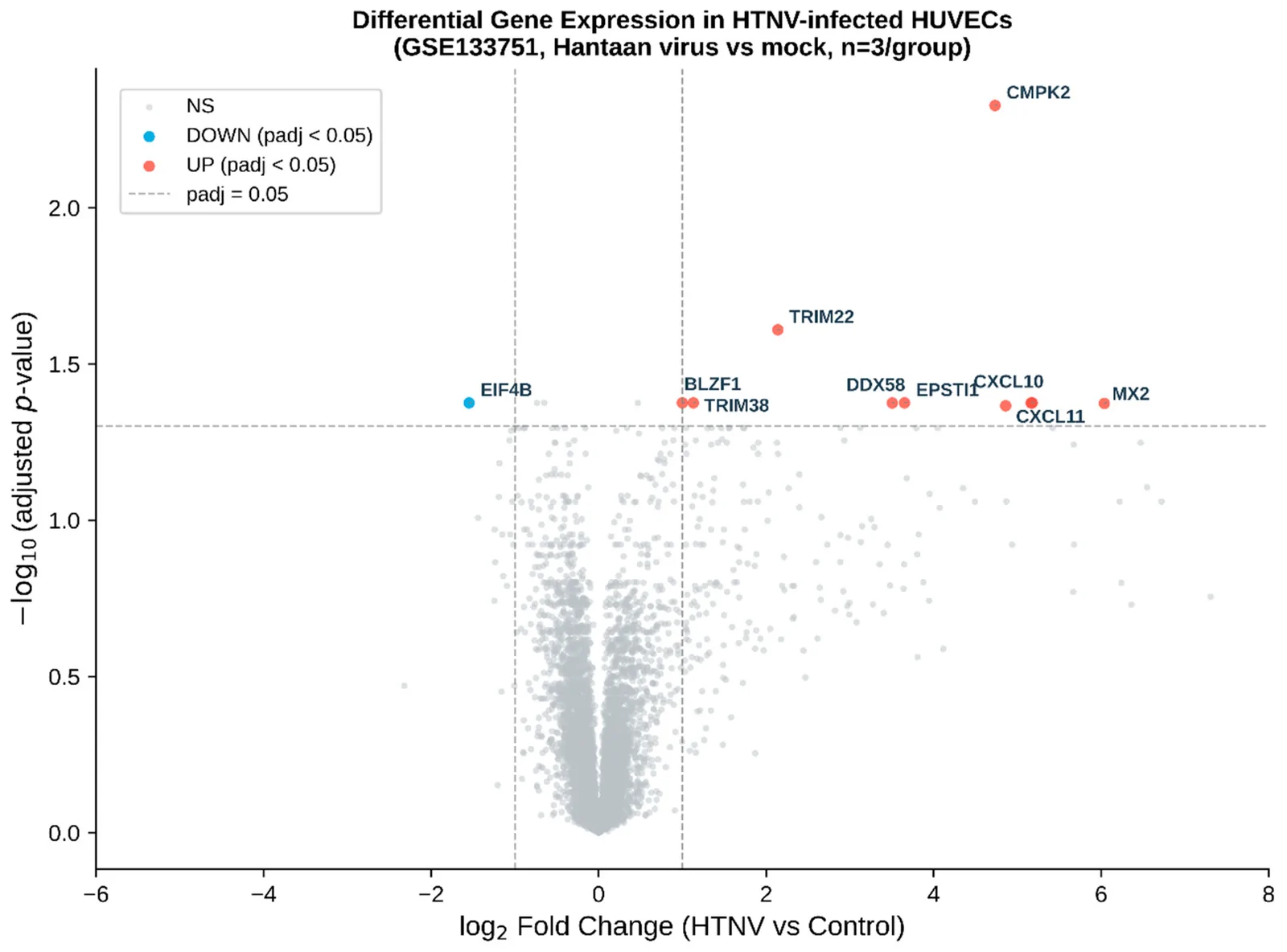
Volcano plot of differentially expressed genes in HTNV-infected HUVECs. Each point represents one gene; the x-axis shows log_2_ fold change (HTNV vs. mock), and the y-axis shows −log_10_(BH-adjusted *p*-value). Red: upregulated DEGs (padj < 0.05, |log_2_FC| ≥ 1); blue: downregulated DEGs; grey: non-significant. Selected ISGs are labelled. Dashed lines indicate padj = 0.05 (horizontal) and |log_2_FC| = 1 (vertical). Data are from GEO accession GSE133751 (*n* = 3 per group) [14].

RNA-seq data from HTNV-infected human umbilical vein endothelial cells (HUVECs) were obtained from the Gene Expression Omnibus (GEO accession GSE133751; *n* = 3 infected and *n* = 3 mock, 72 h post-infection, MOI = 1) [14]. Raw count matrices were processed using DESeq2 (R (4.4)/Bioconductor). At the stringent Benjamini–Hochberg threshold (adjusted *p* < 0.05, |log_2_FC| ≥ 1), 358 genes were differentially expressed (293 upregulated, 65 downregulated). At a nominal threshold (*p* < 0.05, |log_2_FC| ≥ 1), 543 genes were identified (399 upregulated, 144 downregulated). The network and enrichment analyses reported in Section 3.2, Section 3.3 and Section 3.4 were seeded with the 203 genes that met the nominal criteria (*p* < 0.05, |log_2_FC| ≥ 1) in the initial RPKM-based analysis, and are retained here for continuity with the drug-prioritisation and ODE stages that were built upon them. Because this gene set predates the corrected differential-expression analysis, it is referred to throughout as the nominally significant gene set rather than as a validated DEG set; it was chosen to capture the broader IFN-I response consistent with the original study [14], which reported 788 DE mRNAs at q < 0.05. Networks rebuilt from the corrected gene sets at four inclusion thresholds and at two STRING confidence thresholds are reported in Supplementary Tables S2 and S3. The core interferon hubs (ISG15, CXCL10, IRF1, STAT1, IFI35, MX1 and DDX58) are recovered among the top-ranked nodes of every one of these reconstructions, although the composition of the remainder of the top 15 varies with the gene set used. The volcano plot (Figure 1) displays −log_10_(BH-adjusted *p*-value) on the y-axis. Pathway enrichment analysis was performed using Enrichr [15] against the Reactome and Gene Ontology Biological Process (GO:BP) databases. Complete DESeq2 results for all 16,510 genes tested are provided in Supplementary Table S1. The background gene universe for enrichment analysis consisted of all genes tested in the DESeq2 analysis (*n* = 16,510 after low-count filtering), and enrichment *p*-values were adjusted for multiple testing using the Benjamini–Hochberg method as implemented in Enrichr.

DESeq2 version 1.46.0 (R/Bioconductor) was used with a design formula of ~condition (HTNV vs. mock). Preprocessing removed genes with fewer than 10 total counts across all samples. Normalisation was performed using the median of ratios method on raw count data extracted from the Gene Expression Omnibus database. Sample-level quality control was performed using principal component analysis (PCA) on variance-stabilised transformed counts (Figure S1), which confirmed clear separation between HTNV-infected and mock samples along PC1. At the stringent threshold of BH-adjusted *p* < 0.05 and |log_2_FC| ≥ 1, 358 genes were identified as differentially expressed (293 upregulated, 65 downregulated). At a relaxed threshold of FDR < 0.05 and |log_2_FC| ≥ 0.5, 784 genes were identified, closely matching the 788 differentially expressed mRNAs reported in the original publication [14]. The discrepancy between our initial analysis (11 FDR-significant genes) and the corrected analysis (358 genes) was traced to the use of RPKM values as input to DESeq2 in the initial pipeline; the corrected analysis using raw counts reconciles with the original publication. The complete DESeq2 output for all 16,510 genes tested is provided in Supplementary Table S1.

### 2.2. PPI Network Construction

A protein–protein interaction (PPI) network was constructed by querying the STRING database (v12.0) [16] with all 203 nominally significant genes as seed proteins. Only interactions with a combined confidence score ≥ 0.4 were retained. The resulting network comprised 176 nodes and 3210 edges (mean degree 36.5; clustering coefficient 0.71). Network topology was computed using the NetworkX Python library (v3.x). STRING combined confidence scores integrate evidence from the experiments, curated-database, co-expression, genomic-neighbourhood, gene-fusion, co-occurrence and text-mining channels. STRING edges therefore indicate functional association, which does not always mean physical binding. Of the 203 seed proteins, 176 had at least one interaction at the applied confidence threshold; the remaining 27 were isolated and were excluded from the network and from the centrality calculations. To assess the dependence of the ranking on the confidence threshold, the network was also rebuilt from the corrected gene set at combined confidence scores of 0.4 and 0.7, yielding 296 and 216 connected nodes respectively (Supplementary Table S2); the Spearman correlation between composite scores at the two thresholds was 0.60, and the core interferon hubs remained among the top-ranked nodes at both.

### 2.3. Network Centrality and Hub Ranking

Three standard centrality metrics were computed for each node: degree centrality (*C_D_*), betweenness centrality (*C_B_*), and closeness centrality (*C_C_*). A composite network centrality (CNC) score was defined as:

(1) $${\mathrm{CNC}}_{i}=\frac{1}{3}\left({\hat{C}}_{D,i}+{\hat{C}}_{B,i}+{\hat{C}}_{C,i}\right)$$

where ${\hat{C}}_{X,i}$ denotes the min–max normalised value of centrality metric *X* for node *i*. Nodes were ranked in descending order of CNC score, and the top 15 were designated as hub proteins for downstream druggability analysis.

The equal weighting of the three centrality measures and the selection of the top 15 hubs were chosen as heuristic defaults. To assess the robustness of hub rankings to these choices, we performed (i) threshold sensitivity analysis by varying the DEG inclusion criterion (FDR < 0.05 with |log2FC| ≥ 0.5, ≥1, and ≥2; nominal *p* < 0.05 with |log2FC| ≥ 1) and (ii) leave-one-sample-out analysis by removing each of the six samples in turn, re-running DESeq2, reconstructing the STRING network, and recomputing centrality rankings (Supplementary Table S3). Across all threshold variations, Spearman rank correlations with the reference ranking exceeded 0.88. Across all leave-one-out iterations, rank correlations exceeded 0.93, indicating that hub rankings are robust to both threshold choice and sample removal. The reference ranking for these comparisons is the corrected FDR-based reconstruction reported in Supplementary Table S2. These analyses establish the stability of the centrality ranking to gene-selection and sampling choices; they are not a validation of the nominally significant seed set used to build the primary network of Section 2.2.

### 2.4. Druggability Assessment

Druggability of the top 15 hub proteins was assessed using two complementary approaches. First, each protein was queried against the Open Targets Platform (v24.x) [17] via its GraphQL API, retrieving tractability scores for small-molecule and antibody modalities (range 0–4). Second, a literature search was conducted in PubMed using gene-specific queries combined with the terms “drug target”, “inhibitor”, “antiviral”, and “hantavirus”. This search was exploratory rather than a systematic review: records were screened by title and abstract for evidence that a compound modulates the queried protein or its pathway, and no formal inclusion protocol or duplicate screening was applied.

### 2.5. Drug Repurposing Framework

Repurposing candidates were identified by cross-referencing druggable hub proteins with approved and investigational compounds in DrugBank, ChEMBL, and the primary literature. Candidates were stratified into four priority tiers:

1. FDA-approved antivirals with direct antiviral mechanism or prior use in viral haemorrhagic fevers.
2. FDA-approved immunomodulators targeting hub proteins in the JAK–STAT pathway.
3. Investigational agents with direct hantavirus evidence in cell culture or animal models.
4. Investigational broad-spectrum antivirals or biologics targeting hub proteins.

As a caveat: a compound’s tier reflects its regulatory status and the kind of evidence available for it and is not strictly a prediction of efficacy against hantavirus. Approval for another indication is not in itself evidence of anti-hantaviral activity, and a discontinued compound may carry stronger hantavirus-specific mechanistic evidence than an approved one. We did not attempt a formal multidimensional evidence score, because the underlying evidence for most of these compounds in hantavirus systems is too sparse to support quantitative weighting.

### 2.6. Molecular What-If ODE Model

To assess the molecular consequences of drug interventions on the IFN-I signalling cascade, we developed a phenomenological ODE model comprising six state variables representing key nodes of the pathway.

The six state variables were selected to represent the minimal mechanistic scaffold capable of capturing the key therapeutic axes identified by the network analysis: viral replication (V), pattern recognition (R, RIG-I/DDX58), transcription factor activation (I, IRF1/IRF3), JAK-STAT signalling (S, STAT1 phosphorylation), ISG effector function (G, composite ISG expression including MX1, OAS1, ISG15, IFIT1), and inflammatory chemokine output (C, CXCL10). This reduction retains the principal signalling cascade (RIG-I → IRF1 → IFN-beta → JAK/STAT → ISGs → viral clearance) and the inflammatory branch (RIG-I → IRF1 → CXCL10) while omitting auxiliary regulatory interactions such as STAT1 → IRF1 feedback, NF-kB crosstalk, and adaptive immune responses. The consequences of these omissions, particularly the STAT1 → IRF1 feedback, are explored through structural sensitivity analysis (Section 4.7).

- *V*(*t*): normalised viral load (range 0–1);
- *R*(*t*): RIG-I/DDX58 activity;
- *I*(*t*): IRF1/IRF3 transcription factor activity;
- *S*(*t*): STAT1 phosphorylation level;
- *G*(*t*): composite ISG expression (ISG15, MX1, OAS1, IFIT1, GBP1);
- *C*(*t*): CXCL10 secretion level.

The ODE system is defined as:

(2) $$\begin{array}{ll} \frac{\mathrm{dV}}{\mathrm{dt}} & =r_{V}\cdot V\left(1-V\right)-k_{\mathrm{MX}1}\cdot G\cdot V \end{array}$$

(3) $$\begin{array}{ll} \frac{\mathrm{dR}}{\mathrm{dt}} & =k_{R}\cdot \frac{V^{n}}{K_{R}^{n}+V^{n}}-d_{R}\cdot R \end{array}$$

(4) $$\begin{array}{ll} \frac{\mathrm{dI}}{\mathrm{dt}} & =k_{I}\cdot R-d_{I}\cdot I \end{array}$$

(5) $$\begin{array}{ll} \frac{\mathrm{dS}}{\mathrm{dt}} & =\frac{k_{S}\cdot I}{1+k_{\mathrm{JAK}}}-d_{S}\cdot S \end{array}$$

(6) $$\begin{array}{ll} \frac{\mathrm{dG}}{\mathrm{dt}} & =k_{G}\cdot S-d_{G}\cdot G \end{array}$$

(7) $$\begin{array}{ll} \frac{\mathrm{dC}}{\mathrm{dt}} & =k_{C}\cdot I-d_{C}\cdot C \end{array}$$

Viral replication (Equation (2)) follows logistic growth suppressed by ISG effectors (*k*_MX1_, representing MX1-like antiviral activity). RIG-I activation (Equation (3)) follows Hill kinetics with coefficient *n* = 2, capturing the cooperative sensing of viral RNA. IRF1 (Equation (4)) is activated by RIG-I and drives both STAT1 phosphorylation (Equation (5), via the IFN-*β* autocrine loop) and CXCL10 secretion (Equation (7)) directly. STAT1 drives ISG expression (Equation (6)). The JAK inhibitor term *k*_JAK_ reduces STAT1 phosphorylation without affecting the IRF1–CXCL10 axis, consistent with the known pharmacology of baricitinib and ruxolitinib. Because this term is saturating, a value of 0.8 corresponds to a 44% reduction in STAT1 input rather than 80%. Baseline parameter values are listed in Table 1.

Initial conditions were set to *V*(0) = 0.05 (small viral inoculum) and *R*(0) = *I*(0) = *S*(0) = *G*(0) = *C*(0) = 0 (uninfected host baseline). The system was integrated over 14 days using the Runge–Kutta RK45 method as implemented in SciPy [18] (relative tolerance 10^−8^).

The model time axis (0–14 days) represents the acute phase of viral replication in endothelial cells and does not map directly to the clinical timeline of human hantavirus disease, which includes an incubation period and complex temporal relationships between viral replication, innate immunity, and symptomatic presentation. The early (*t* = 0) and delayed (t = 3 days) intervention timepoints should be interpreted as model perturbations representing pre-emptive versus post-establishment treatment, rather than precise clinical timing recommendations.

#### 2.6.1. Parameter Estimation and Biological Rationale

The fifteen parameters of the what-if ODE model (Table 1) were not fitted to quantitative time-course data, which are not yet available for Hantaan virus (HTNV) infection of human endothelial cells at the temporal resolution required. Each parameter was assigned a biologically plausible value based on published evidence from hantavirus biology, interferon signalling kinetics, and pharmacological data on the candidate drugs. The model variables are dimensionless (normalised to carrying capacity or steady-state maxima), and parameters are expressed in units of inverse days (d^−1^), consistent with the 14-day simulation horizon that spans the acute phase of hantavirus disease [3]. We provide the rationale for each parameter group below.

Viral replication rate (*r_V_* = 1.2 d^−1^). The logistic growth rate *r_V_* governs viral expansion in the absence of immune pressure. Hantavirus RNA replication kinetics have been characterised in several in vitro systems: Wigren Byström et al. measured Puumala virus (PUUV) transcript accumulation in Vero cells and observed a lag of approximately 9 h followed by rapid exponential amplification, with S- and M-segment transcripts reaching thousand-fold higher copy numbers by days 2–6 post-infection [19]. LaPointe et al. reported comparable replication kinetics for Seoul virus (SEOV) in endothelial cells, with viral RNA detectable within 24 h and plateauing by day 3–5 [20]. Adams et al. developed data-driven mathematical models for Prospect Hill orthohantavirus (PHV) replication and estimated doubling times of 6–12 h during the exponential phase [21], corresponding to intrinsic growth rates of approximately 1.4–2.8 d^−1^. Pathogenic hantaviruses such as HTNV replicate more slowly than non-pathogenic PHV in certain cell types [22], and the delayed IFN induction characteristic of pathogenic strains [23] effectively reduces the net replication rate during the immune-controlled phase. We therefore selected *r_V_* = 1.2 d^−1^, corresponding to a doubling time of approximately 14 h, a growth rate below in vitro range, because of the partial immune pressure in endothelial cells.

RIG-I sensing parameters (*k_R_* = 1.8, *K_R_* = 0.3, *n* = 2.0). The Hill function governing RIG-I induction (Equation (3)) captures the cooperative activation of RIG-I-like receptors by viral RNA. Kell et al. demonstrated that RIG-I-like receptor signalling is essential for innate immune control of HTNV, with loss of this pathway permitting uncontrolled viral replication [4]. The Hill coefficient *n* = 2 reflects the cooperative dimerisation of RIG-I filaments along double-stranded RNA, a well-characterised feature of RIG-I activation that produces a sigmoidal dose–response curve [24]. The half-activation constant *K_R_* = 0.3 was set below the baseline initial viral load (*V*_0_ = 0.05) to ensure that RIG-I induction begins early in infection, consistent with the rapid sensing observed in hantavirus-infected endothelial cells [5]. The induction rate *k_R_* = 1.8 d^−1^ was calibrated so that RIG-I activation reaches half-maximum within approximately 1–2 days, matching the delayed-but-detectable IFN response typically observed at 24–48 h post-infection in HTNV-infected endothelial cells [23]. Importantly, pathogenic hantaviruses actively suppress this sensing: the NSs protein of Andes virus antagonises MAVS signalling downstream of RIG-I [25], and multiple orthohantaviruses inhibit type I IFN induction through distinct viral protein interactions [26,27]. The model captures this evasion implicitly through the moderate *k_R_* value rather than an explicit evasion term.

Protein turnover rates (*d_R_* = 0.4, *d_I_* = 0.5, *d_S_* = 0.6 d^−1^). The decay rates for RIG-I (*d_R_*), IRF1 (*d_I_*), and phosphorylated STAT1 (*d_S_*) reflect protein turnover and deactivation. RIG-I protein stability is regulated by K48- and K27-linked ubiquitination, with RNF20-mediated degradation providing a negative feedback that prevents excessive interferon production [24]. The half-life t½ = ln(2)/*d_R_* ≈ 1.7 days for RIG-I is consistent with the sustained but gradually resolving innate sensing observed during the first week of hantavirus infection. IRF1, an immediate-early transcription factor, has a shorter half-life (t½ ≈ 1.4 days at *d_I_* = 0.5), reflecting the rapid turnover characteristic of inducible transcription factors. STAT1 dephosphorylation (*d_S_* = 0.6, t½ ≈ 1.2 days) is mediated by nuclear phosphatases and SOCS feedback, and the positive feedback loop between IFN-β production and JAK/STAT signalling stabilises nuclear IRF3 and STAT1 in first-responder cells [28]. The ordering *d_S_* > *d_I_* > *d_R_* captures the increasing stability from downstream signalling effectors to upstream sensors.

IFN-β production and JAK-STAT signalling (*k_I_* = 1.5, *k_S_* = 1.2 d^−1^). The rates of IRF1-mediated IFN-β production (*k_I_*) and STAT1 phosphorylation downstream of IFNAR signalling (*k_S_*) were set to produce IFN-β accumulation within 24–48 h and peak STAT1 phosphorylation by day 3–4, consistent with the temporal dynamics of type I IFN responses in hantavirus-infected endothelial cells [8,23]. Czerkies et al. demonstrated that autocrine and paracrine IFNβ–JAK/STAT signalling creates a positive feedback that upregulates RIG-I, PKR, and OAS1A in sensitised cells, effectively shortening the antiviral decision phase [28]; the model captures this feedback through the cascade structure R → I → S → G → V.

JAK inhibition parameter (*k_JAK_* = 0 baseline; *k_JAK_* = 0.8 in scenarios S3, S4, S8). In the baseline model, *k_JAK_* = 0 reflects unhindered JAK1/JAK2 signalling. In drug scenarios, *k_JAK_* = 0.8 represents partial inhibition of STAT1 phosphorylation (Equation (5): dS/dt = *k_S_* · I/(1 + *k_JAK_*) − *d_S_* · S), reducing the effective STAT1 activation rate by approximately 44%. Baricitinib, a JAK1/JAK2 inhibitor, exhibits in vitro IC_50_ values of approximately 1–2 nM for JAK1 and 0.5–1 nM for JAK2 [29,30], achieving near-complete pathway suppression at clinically achievable plasma concentrations. However, the model uses a partial inhibition value rather than complete blockade (*k_JAK_* ≫ 1) for two reasons: (i) tissue penetration in endothelial cells may be submaximal, and (ii) complete JAK blockade would abolish all type I IFN signalling, which is counterproductive given that Ravlo et al. showed JAK inhibitors can facilitate viral propagation [11]. The choice of *k_JAK_* = 0.8 thus represents a moderate, clinically realistic inhibition level.

ISG induction and antiviral effector function (*k_G_* = 1.0, *d_G_* = 0.3, *k_MX_*_1_ = 2.5 d^−1^). The ISG induction rate *k_G_* and decay rate *d_G_* govern the accumulation of interferon-stimulated gene products (represented collectively by the variable G). The half-life t½ = ln(2)/*d_G_* ≈ 2.3 days reflects the sustained expression of ISG mRNAs and proteins, many of which (e.g., MxA, OAS, PKR) remain elevated for several days after IFN induction [6]. The antiviral suppression rate *k_MX_*_1_ = 2.5 d^−1^ quantifies the potency of ISG-mediated viral clearance (Equation (2): −*k_MX_*_1_ · G · V). This term is named for MX1 (MxA in humans), a dynamin-like GTPase that is among the most potently antiviral ISGs against negative-sense RNA viruses. MxA forms oligomeric rings around viral nucleocapsids, physically disassembling replication complexes and blocking transcription [31,32]. MxA has been shown to restrict hantavirus replication specifically [33], and the high *k_MX_*_1_ value relative to *r_V_* ensures that once ISGs accumulate, viral clearance is rapid—consistent with the steep decline in viral load observed after IFN induction in vitro [34].

CXCL10 induction and clearance (*k_C_* = 0.8, *d_C_* = 0.4 d^−1^). CXCL10 (IP-10) is modelled as a downstream product of IRF1 activation (Equation (7): dC/dt = *k_C_* · I − *d_C_* · C), reflecting its transcriptional induction by IRF1 and NF-κB downstream of RIG-I signalling. Jiang et al. reported that serum CXCL10 is markedly elevated in severe hantavirus infection and correlates with disease severity [35]. The decay rate *d_C_* = 0.4 d^−1^ (t½ ≈ 1.7 days) reflects the combined effects of secretion, dilution, DPP4-mediated N-terminal truncation [36], and GAG-dependent scavenging. Meissner et al. observed that CXCL10 levels decay rapidly after initiation of antiviral therapy in chronic HCV infection, with measurable declines within days of viral suppression [37], supporting a relatively short biological half-life. In the anti-CXCL10 scenario (S6), increasing *d_C_* fivefold models enhanced antibody-mediated neutralisation; the fivefold factor is a modelling choice representing a substantial increase in clearance rather than a measured neutralisation rate for any specific antibody.

#### 2.6.2. Drug Perturbation Scenarios

Eight drug scenarios were defined as parameter multipliers applied at either *t* = 0 (early intervention) or *t* = 3 days (late intervention, representing clinical presentation after symptom onset). For two-phase scenarios, the ODE system was integrated separately over [0,*t*_onset_] and [*t*_onset_,14], with the state vector at *t*_onset_ serving as the initial condition for the second phase. The eight scenarios are summarised in Table 2.

These eight scenarios were selected to represent the principal therapeutic axes identified by the network analysis: IFN-beta augmentation (S1, S2), JAK-STAT modulation (S3, S4), RIG-I pathway activation (S5), CXCL10 neutralisation (S6), combination antiviral plus anti-inflammatory therapy (S7), and sequential therapy (S8). The ODE model explores selected pathway-level perturbations suggested by the preceding network analysis; it does not simulate all 12 repurposing candidates (e.g., ribavirin, remdesivir, and favipiravir act through viral RdRp inhibition, which is not represented in the IFN-centric model). The model functions as a mechanistic exploration of the IFN/JAK-STAT/CXCL10 axis, and not as a strict quantitative validation of the complete drug repurposing list.

Outcome metrics computed for each scenario were: (i) peak viral load max*_t_V*(*t*); (ii) viral clearance time (first *t* at which *V* < 0.01); (iii) peak CXCL10 max*_t_C*(*t*); (iv) ISG area under the curve $\int_{0}^{14}G\left(t\right)\;\mathrm{dt}$; and (v) peak STAT1 max*_t_S*(*t*).

## 3. Results

### 3.1. Transcriptomic Landscape of HTNV-Infected Endothelial Cells

Differential expression analysis of GSE133751 identified 358 DEGs at the stringent FDR-corrected threshold (BH-adjusted *p* < 0.05, |log_2_FC| ≥ 1; 293 upregulated, 65 downregulated) and 543 DEGs at the nominal threshold (*p* < 0.05, |log_2_FC| ≥ 1; 399 upregulated, 144 downregulated) in HTNV-infected HUVECs relative to mock-infected controls. The initial RPKM-based analysis identified 11 FDR-significant and 203 nominal DEGs; the corrected raw-count analysis reconciles with the original publication [14] (see Section 2.1). The volcano plot (Figure 1) illustrates the distribution of fold changes and statistical significance. The upregulated programme was dominated by ISGs. The strongest signals included CMPK2, CXCL10, CXCL11, MX2, and DDX58/RIG-I (exact log_2_FC and FDR in Supplementary Table S1). EIF4B, a translation initiation factor, was among the downregulated genes; its modest fold change is biologically plausible but should be interpreted cautiously given the single-condition design. Pathway enrichment of the nominally upregulated genes confirmed strong activation of interferon signalling (Reactome, *p* = 1.3 × 10^−63^) and defence response to virus (GO:BP, *p* = 3.8 × 10^−58^), with concurrent enrichment for ribosomal translation among the downregulated genes.

To address the dependence of pathway inference on the nominal *p* < 0.05 threshold, a rank-based gene set enrichment analysis (GSEA) was performed using the full DESeq2 log2 fold-change distribution for all 16,510 genes tested (Supplementary Table S4). This threshold-free analysis confirmed that the interferon/antiviral response dominates the transcriptional response: 151 pathways were significantly upregulated (padj < 0.05), with ‘Response to virus’ (NES = 2.43, padj = 2.7 × 10^−29^), ‘Defense response to virus’ (NES = 2.45, padj = 5.3 × 10^−27^), and ‘Type I interferon-mediated signalling pathway’ (NES = 2.29, padj = 2.6 × 10^−10^) among the top-ranked pathways (Figure S2). All key hub genes (ISG15, STAT1, IRF1, CXCL10, MX1, DDX58) appeared in the leading edge of these pathways, providing threshold-independent validation of the network architecture.

### 3.2. Active Pathways and Network Architecture

#### 3.2.1. IFN-I Induction Axis

Hantavirus genomic RNA is sensed by the cytosolic helicase RIG-I (DDX58) and, to a lesser extent, MDA5 (IFIH1), both of which are strongly upregulated in infected HUVECs (centrality ranks 11 and 23, respectively). Upon RNA binding, RIG-I undergoes conformational activation and signals through the mitochondrial adaptor MAVS, triggering downstream phosphorylation of IRF3 and IRF7 and nuclear translocation of the IRF3 dimer, which drives de novo IFN-*β* transcription [4,5]. The long non-coding RNA NEAT1 has been shown to act as a positive feedback amplifier of this axis in hantavirus-infected cells [5].

#### 3.2.2. JAK–STAT Axis and ISG Effector Layer

Secreted IFN-*β* binds the IFNAR1/IFNAR2 receptor complex and activates JAK1 and TYK2, leading to phosphorylation of STAT1 and STAT2. The phosphorylated STAT1–STAT2–IRF9 heterotrimer (ISGF3) translocates to the nucleus and drives transcription of ISGs through interferon-stimulated response elements (ISREs) [38]. The ISG effector layer identified in our network includes:

- ISG15 (centrality rank 1, degree 100): a ubiquitin-like modifier that is conjugated to hundreds of host and viral proteins (ISGylation), broadly suppressing viral replication and modulating immune signalling.
- MX1 (centrality rank 7, degree 88): a dynamin-like GTPase that physically traps viral ribonucleoprotein complexes, preventing transcription of the viral genome.
- OAS1 (centrality rank 49, degree 82): activates RNase L upon binding double-stranded RNA, leading to degradation of both viral and cellular RNA and triggering apoptosis in infected cells.
- IFIT1 (centrality rank 25, degree 82): sequesters viral 5′-triphosphate RNA and inhibits eIF3-dependent translation initiation.
- GBP1 (centrality rank 8, degree 84): a guanylate-binding protein that disrupts viral membranes and activates the NLRP3 inflammasome.
- RSAD2/Viperin (centrality rank 50, degree 79): remodels lipid droplets and produces the antiviral nucleotide ddhCTP, inhibiting viral RdRp.

#### 3.2.3. CXCL10 Chemokine Axis

IRF1 (centrality rank 2, degree 92) is a transcription factor activated both by RIG-I–MAVS signalling and by STAT1, creating a feed-forward loop that amplifies ISG transcription. Critically, IRF1 also directly drives the expression of CXCL10 (IP-10) and CXCL11, potent chemoattractants for CXCR3-expressing T cells and NK cells [7]. Elevated CXCL10 levels correlate with disease severity in HFRS patients and are thought to contribute to the T-cell-mediated vascular leakage that defines severe disease [7,8]. In our model formulation, CXCL10 production is linked directly to primary RIG-I–IRF1 activation (Equation (7)). This design isolates TF-mediated chemokine release triggered directly by viral sensing from downstream interferon feedback. Under this parameterisation, JAK inhibition selectively dampens STAT1-dependent ISGs without impairing the upstream IRF1-driven chemokine branch, allowing us to evaluate the consequences of uncoupling viral clearance from chemokine induction.

#### 3.2.4. NF-*κ*B Axis

Parallel to the IRF3/IRF7 pathway, MAVS activates TRAF3 and TRAF6, which phosphorylate the IKK complex and release NF-*κ*B from its inhibitor I*κ*B. Nuclear NF-*κ*B drives transcription of pro-inflammatory cytokines (TNF-*α*, IL-6, IL-8) and co-stimulatory molecules, amplifying the inflammatory response. Several NF-*κ*B target genes (BIRC3, TNFAIP3, VCAM1, ICAM1) are present in the DEG set, consistent with endothelial activation.

#### 3.2.5. Protein Quality Control Axis

Valosin-containing protein (VCP/p97), an AAA-ATPase involved in endoplasmic-reticulum-associated degradation (ERAD) and autophagy, was recently shown to interact directly with the hantavirus nucleocapsid protein and to be required for efficient viral replication [39]. Although VCP is not among the top-15 hubs by CNC score (it is not a DEG in the transcriptomic dataset), it represents a mechanistically validated host-dependency factor and is included as a Tier 3 drug target.

**Figure 2:**
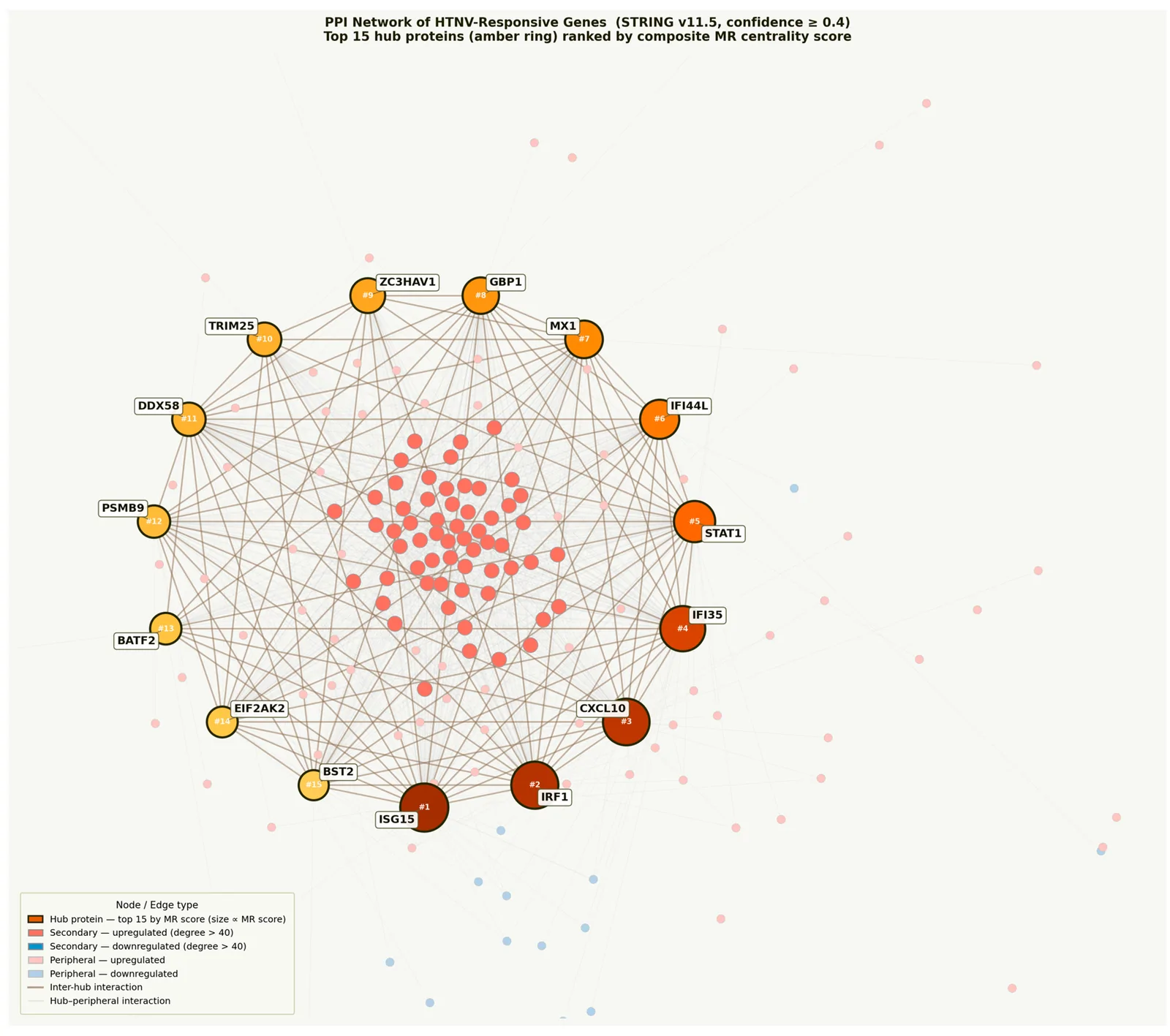
PPI network of HTNV-responsive genes. Nodes represent proteins encoded by DEGs; edges represent STRING interactions (confidence ≥ 0.4). Node size is proportional to degree centrality. The top 15 hub proteins (highest composite CNC score) are highlighted in amber and labelled; rank badges (#1–#15) are shown inside each hub node. Amber-to-brown gradient encodes CNC score magnitude. Salmon/steel-blue nodes: secondary proteins (degree > 40); pale tints: peripheral proteins. Network constructed from STRING v12.0 [16].

The PPI network with hub proteins highlighted is shown in Figure 2. The network displays a densely connected core of ISGs centred on *STAT1*, *IRF1*, and *ISG15*, consistent with the known architecture of the IFN-I regulatory network.

### 3.3. Druggable Hub Proteins

Open Targets tractability analysis identified six of the 15 hub proteins as druggable (score ≥ 1; Table 3). CXCL10 achieved the highest tractability score (4), reflecting clinical-stage evidence for anti-CXCL10 biologics in inflammatory diseases. STAT1, MX1, GBP1, and DDX58 each scored 2, indicating strong preclinical or early clinical evidence. ISG15 scored 1, reflecting biochemical tractability without advanced clinical validation. OAS1 also scored 1 but ranks 49th by CNC score and therefore lies outside the top-15 hub set; it enters the repurposing analysis as an ISG effector, not as a hub.

### 3.4. Drug Repurposing Candidates

**Figure 3:**
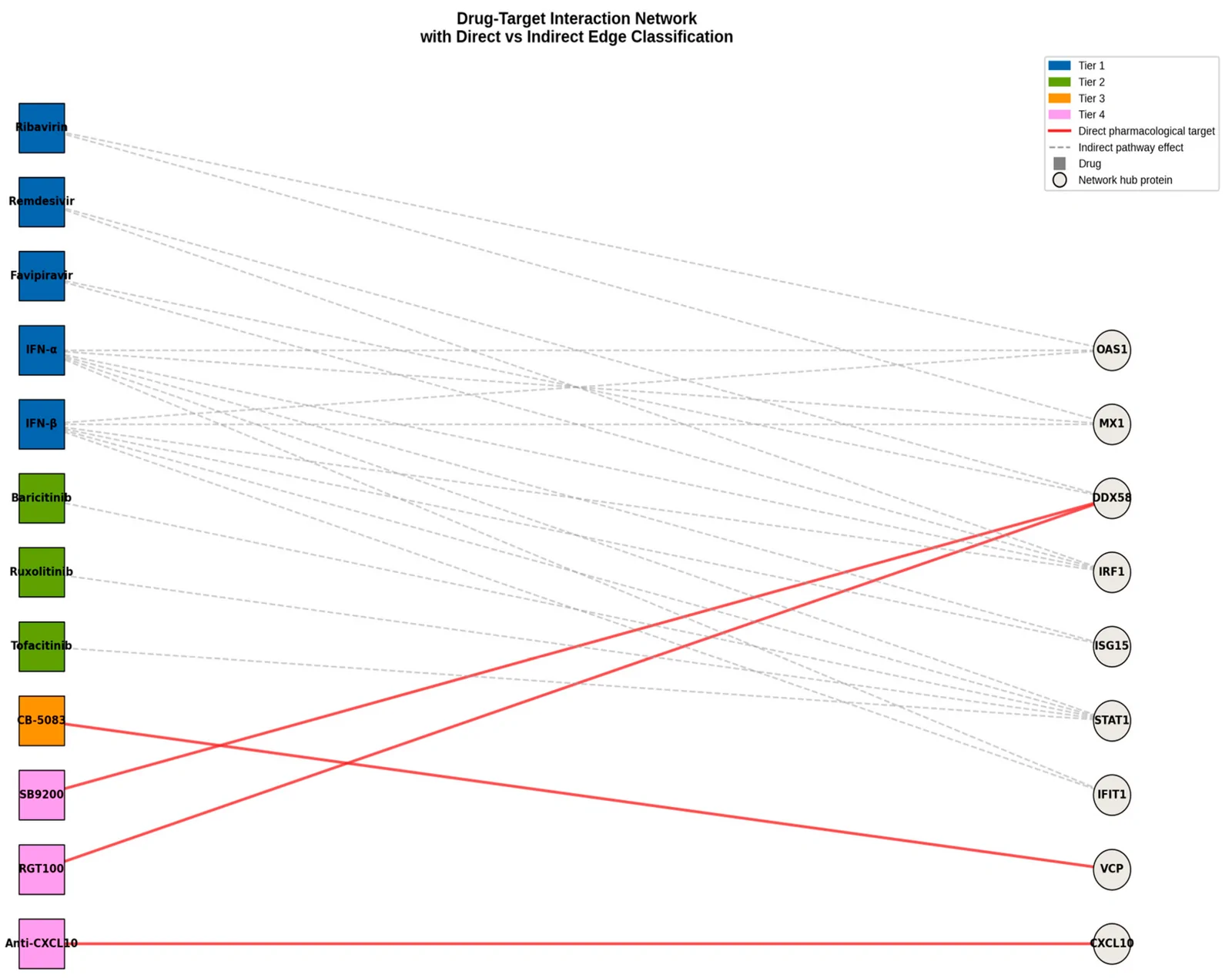
Bipartite drug–target interaction network with direct vs. indirect edge classification. Left nodes (squares) represent the 12 drug repurposing candidates, coloured by priority tier (Tier 1–4). Right nodes (circles) represent the proteins targeted by the candidate compounds. Most are network hubs from Table 3; OAS1, IFIT1, and VCP fall outside the top-15 hub set and are included as literature-added, mechanistically validated candidates rather than as network-derived hubs. Solid red edges indicate direct pharmacological targets (e.g., anti-CXCL10 antibody binding CXCL10, RIG-I agonists binding DDX58, CB-5083 binding VCP). Dashed grey edges indicate indirect pathway effects (e.g., remdesivir reducing viral RNA, which indirectly reduces DDX58/IRF1 activation; IFN-β triggering downstream ISG expression through IFNAR). Node size is proportional to composite network centrality (CNC) score.

**Figure 4:**
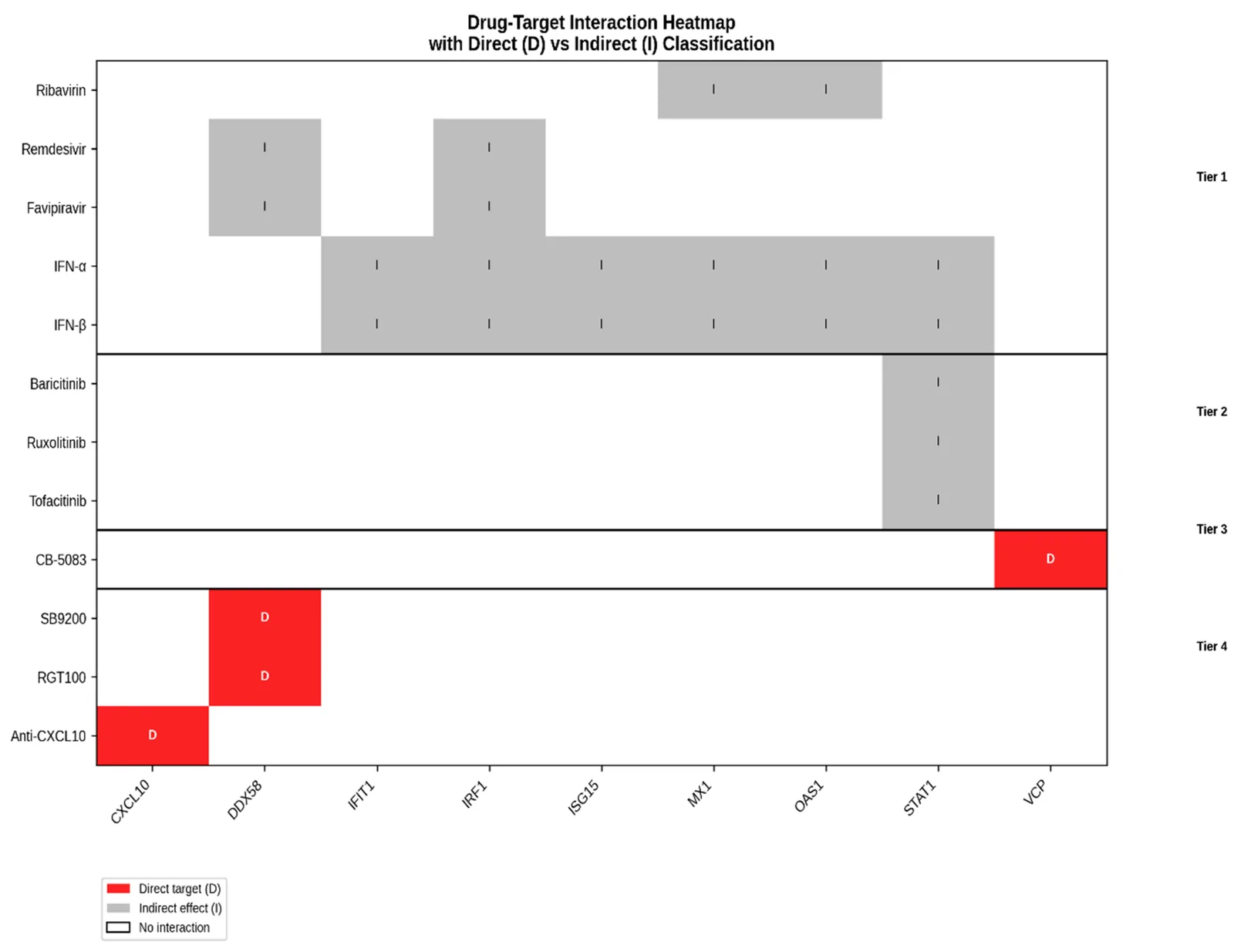
Drug–target interaction heatmap with direct (D) vs. indirect (I) classification. Rows represent the 12 drug repurposing candidates (grouped by priority tier); columns represent the targeted network proteins, of which OAS1, IFIT1, and VCP lie outside the top-15 hub set (see Figure 3 caption). Red cells with “D” denote direct pharmacological targets; grey cells with “I” denote indirect pathway effects; white cells indicate no interaction. This distinction separates pharmacological binding relationships from downstream functional consequences.

Cross-referencing druggable hub proteins with approved and investigational compounds yielded 12 repurposing candidates (Table 4). The drug–target interaction network (Figure 3) and heatmap (Figure 4) visualise the coverage of hub proteins by each candidate. The bipartite network comprised 23 nodes (12 drugs + 11 targeted hub proteins) and 35 edges (density 0.27). *IRF1* was the most targeted hub (9/12 drugs), reflecting its central position downstream of both RIG-I and IFN-*β* signalling. *STAT1* was targeted by six agents. Direct-acting antivirals (remdesivir, favipiravir) inhibit the viral RdRp; in these network mappings (Figure 3), they are connected to DDX58 and IRF1 to represent their indirect functional attenuation of viral RNA-mediated pattern recognition receptor sensing.

The drug-target network (Figure 3) and heatmap (Figure 4) distinguish direct pharmacological targets (solid edges, red cells; e.g., anti-CXCL10 antibody binding CXCL10, RIG-I agonists binding DDX58, CB-5083 binding VCP) from indirect pathway effects (dashed edges, grey cells; e.g., remdesivir reducing viral RNA, which indirectly reduces DDX58/IRF1 activation; IFN-beta triggering downstream ISG expression through IFNAR). Because the network mixes direct and indirect interactions, centrality on it can merge pharmacological potency with pathway position; the classification in Figure 3 and Figure 4 makes that distinction visible. The drug prioritisation stage (Table 4) should be interpreted as identifying pathway-level intervention points rather than confirming direct drug–protein binding for all shown edges. The candidate set is also not derived from the network alone. Ten of the twelve candidates act on proteins within the top-15 hub set, whereas OAS1 (CNC rank 49) and IFIT1 (rank 25) enter as ISG effectors and VCP enters purely from the literature, being a mechanistically validated host-dependency factor that is not differentially expressed in this dataset. We distinguish these literature candidates from the network ones in the analysis.

Tier 1—FDA-Approved Antivirals. *Ribavirin* is a nucleoside analogue with broad-spectrum antiviral activity used off-label for HFRS and HCPS [40]. *Remdesivir* has demonstrated inhibitory activity against Sin Nombre and Puumala hantaviruses in primary human endothelial cells [41]. *Favipiravir* synergises with ribavirin in reducing Hantaan virus titres in cell culture [42]. *Interferon-α* and *interferon-β* potently induce ISG expression and have shown anti-hantaviral activity in combination with ribavirin [40,43].

Tier 2—FDA-Approved Immunomodulators. JAK1/JAK2 inhibitors baricitinib and ruxolitinib, and the JAK1/JAK3 inhibitor tofacitinib, suppress STAT1 phosphorylation and downstream ISG transcription [44]. Their rationale for use in hantavirus infection is to attenuate the excessive interferon-driven CXCL10-associated inflammatory signalling that contributes to vascular leakage in severe disease. However, their use carries a critical caveat: JAK inhibitors can facilitate viral propagation if administered during the early replication phase [11].

Tier 3—Investigational, Hantavirus-Specific. *CB-5083* is a first-in-class inhibitor of VCP/p97 that reduced hantavirus titres by >2 log_10_ in cell culture [39], establishing VCP as a validated host-directed antiviral target.

The RIG-I agonist SB9200 (vesatolimod) was discontinued in Phase 2 due to hepatotoxicity concerns. RGT100, an alternative RIG-I agonist currently in Phase 1, may offer a safer profile but requires further clinical evaluation. These toxicity risks should be carefully weighed against the potential antiviral benefits predicted by the model.

Tier 4—Investigational, Broad-Spectrum. SB9200 and RGT100 are small-molecule RIG-I agonists that reached early clinical testing. Development of SB9200 (inarigivir soproxil) was halted after hepatotoxicity and a patient death in a Phase 2b chronic hepatitis B trial, so it is retained here as a mechanistic exemplar of RIG-I agonism instead of as a near-term clinical candidate. An anti-CXCL10 monoclonal antibody in preclinical/Phase 1 development targets the CXCL10 inflammation axis directly [7].

### 3.5. Molecular What-If Analysis

**Figure 5:**
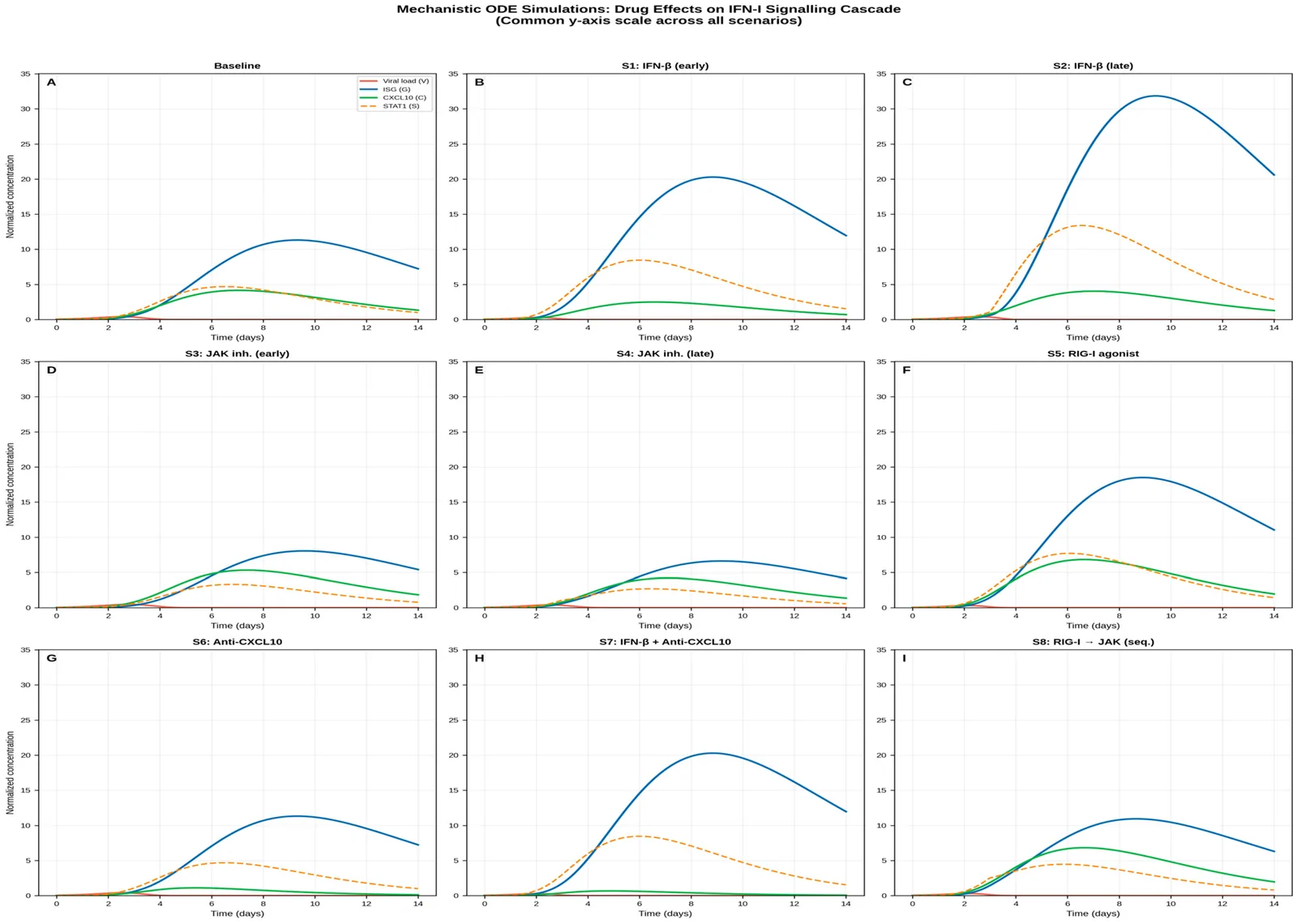
Mechanistic ODE simulations: drug effects on the IFN-I signalling cascade. Each panel shows the time course of viral load (red), ISG composite (blue), CXCL10 (green), and STAT1 phosphorylation (orange dashed) over 14 days. All panels share a common y-axis scale (0–35) to enable direct visual comparison of absolute magnitudes across scenarios. (**A**) Baseline. (**B**) S1: early IFN-β (t = 0). (**C**) S2: late IFN-β (t = 3d). (**D**) S3: early JAK inhibitor (t = 0). (**E**) S4: late JAK inhibitor (t = 3d). (**F**) S5: RIG-I agonist. (**G**) S6: anti-CXCL10 mAb. (**H**) S7: IFN-β + anti-CXCL10. (**I**) S8: RIG-I agonist → JAK inhibitor (sequential).

The ODE model was integrated over 14 days for all eight drug scenarios and the drug-free baseline. Figure 5 shows the time courses of viral load (*V*), ISG composite (*G*, normalised), CXCL10 (*C*, normalised), and STAT1 (*S*, normalised) for each scenario. Summary metrics are reported in Table 5.

Figure 5 uses a common y-axis scale across all scenarios for each variable, enabling direct visual comparison of absolute magnitudes between scenarios. The y-axis range (0–35) reflects the maximum value reached by any variable in any scenario (peak STAT1 in S2). Table 5 reports the unnormalised model values; the apparent discrepancy between the figure and Table 5 values greater than 1 has been resolved by adopting the common-scale presentation.

Early IFN-β (S1) achieves the fastest viral clearance. Tripling the STAT1 phosphorylation rate at t = 0 reduces peak viral load by 27% (0.294 vs. 0.402) and accelerates clearance by 0.8 days relative to baseline. The ISG AUC increases by 85% (164.0 vs. 88.8), reflecting the amplified antiviral effector response. CXCL10 is reduced by 40% relative to baseline (2.495 vs. 4.163), because faster viral clearance shortens the duration of IRF1 activation. This CXCL10 reduction is specific to the present topology: when STAT1-dependent CXCL10 induction is included, the same intervention raises CXCL10 instead (Section 4.7).

Late IFN-β (S2) provides modest benefit. When IFN-β is administered at t = 3 days (after peak viral load), viral clearance improves only marginally (4.0 vs. 4.3 days). The ISG AUC is paradoxically higher (243.0) because the prolonged viral replication phase generates more IRF1 activity before the drug takes effect, and the subsequent IFN boost then drives a large but belated ISG response.

Early JAK inhibition (S3) is counterproductive. Applying k_JAK_ = 0.8 at t = 0 reduces STAT1 phosphorylation and ISG expression (AUC 62.0 vs. 88.8), impairing the antiviral effector response. Peak viral load increases to 0.470 and clearance is delayed to 4.9 days. Critically, CXCL10 rises to 5.315 (vs. 4.163 at baseline) because prolonged viral replication drives more IRF1 activity, which feeds CXCL10 independently of STAT1. The direction of this result matches the observation of Ravlo et al. [11] that JAK inhibition can facilitate viral propagation, although the mechanisms differ: their effect arises from the loss of interferon-stimulated gene expression in uninfected bystander cells, which a single-compartment model cannot represent.

Late JAK inhibition (S4) is safer but provides limited benefit. Delaying JAK inhibition to *t* = 3 days preserves the early antiviral ISG response. Viral clearance is only marginally delayed (4.5 vs. 4.3 days), and STAT1 is reduced after the drug onset. However, CXCL10 remains virtually unchanged (4.217 vs. 4.163). In this model framework, where chemokine output reflects upstream IRF1 activity rather than secondary STAT1 signalling, JAK inhibition alone cannot extinguish the inflammatory signal once primary viral sensing has occurred.

RIG-I agonists (S5) accelerate clearance but amplify CXCL10. Boosting RIG-I sensing (*k_R_* × 2.5) reduces peak viral load (0.310) and accelerates clearance (3.6 days), comparable to early IFN-*β*. However, the enhanced RIG-I–IRF1 signalling also drives a large CXCL10 response (6.822 vs. 4.163 at baseline), representing substantial CXCL10-associated immunopathology risk. This trade-off suggests that RIG-I agonists may warrant investigation as a combination therapy with CXCL10 neutralisation in severe disease.

Anti-CXCL10 mAb (S6) selectively suppresses CXCL10 output. Increasing the CXCL10 decay rate (*d_C_* × 5.0) reduces peak CXCL10 by 73% (1.109 vs. 4.163) without affecting viral dynamics (clearance 4.3 days, identical to baseline). Because CXCL10 does not feed back onto any other state variable, this invariance is a structural property of the model rather than an emergent result. Within this framework, CXCL10 neutralisation acts only on the CXCL10 axis and must be combined with an antiviral agent for therapeutic benefit.

Combination S7 (IFN-*β* + anti-CXCL10) is the most favourable profile among the eight simulated scenarios. Scenario 7 achieves the best overall profile: fastest viral clearance (3.5 days, tied with S1), lowest peak CXCL10 (0.671, an 84% reduction from baseline), and high ISG AUC (164.0). By simultaneously boosting antiviral ISG expression and neutralising the CXCL10 signal, this combination addresses both arms of the therapeutic dilemma. The antiviral metrics of S7 are identical to those of S1 by construction, so the incremental value of the combination lies entirely on the chemokine axis. This combination represents a rational mechanistic hypothesis for experimental testing rather than definitive evidence of therapeutic superiority. Its chemokine advantage is also topology-dependent and does not persist when STAT1-dependent CXCL10 induction is added to the model (Section 4.7).

Sequential RIG-I agonist → JAK inhibitor (S8) does not suppress CXCL10. Despite the sequential design intended to boost early antiviral immunity and then dampen late inflammation, CXCL10 remains elevated (6.825) throughout the simulation. The JAK inhibitor applied at t = 3 days reduces STAT1 but leaves the IRF1–CXCL10 axis undisturbed, and the IRF1 activity induced by the agonist in the first phase is already established.

## 4. Discussion

### 4.1. The IFN-I Axis as the Primary Therapeutic Landscape

The transcriptomic and network analyses converge on the type I interferon signalling axis as the dominant host response to HTNV infection. The top-ranked hub proteins (ISG15, IRF1, CXCL10, STAT1, MX1, DDX58) are all components of this pathway. This is consistent with prior studies showing that hantavirus infection induces a strong but delayed IFN response, and that ISG induction correlates with both viral control and CXCL10-associated inflammatory signalling [8,14]. As discussed in the introduction, the dual role of the IFN-I axis creates a therapeutic dilemma that cannot be resolved by targeting a single node: any intervention that suppresses IFN signalling risks impairing antiviral immunity, while any intervention that amplifies it risks CXCL10-driven inflammation and viral diffusion.

Our ODE simulations make this dilemma quantitatively explicit. Early IFN-*β* (S1) achieves the best antiviral outcome but does not eliminate CXCL10 (peak 2.495 vs. 4.163 at baseline). RIG-I agonists (S5) achieve comparable antiviral efficacy but drive CXCL10 to 6.822, a 64% increase over baseline. The only strategy that resolves the dilemma is combination therapy targeting both arms simultaneously (S7).

### 4.2. Timing Is Everything: The JAK Inhibitor Paradox

The timing analysis illustrates a key advantage of the dynamic model over static network analysis. The comparison of early and late interventions shows that the same drug can produce qualitatively different effects depending on when it is administered relative to the viral replication cycle. This principle, while well established in pharmacology, is made explicit and mechanistically interpretable through the ODE framework.

The most striking finding of the what-if analysis is the qualitative difference between early (S3) and late (S4) JAK inhibition. Early JAK inhibition is counterproductive: it delays viral clearance by 14% and paradoxically elevates CXCL10 by 28% relative to baseline. This result is mechanistically explained by the IRF1–CXCL10 axis: JAK inhibitors suppress STAT1-driven ISG transcription but do not affect IRF1 activity, which is driven by RIG-I upstream of the JAK–STAT cascade. When ISG-mediated viral suppression is impaired, viral replication continues longer, driving more RIG-I and IRF1 activity, and therefore more CXCL10. This is consistent in direction with Ravlo et al. [11], but it is not mechanistically equivalent, since their pro-viral effect is driven by loss of interferon-stimulated gene expression in uninfected bystander cells.

This prediction should be interpreted as a topology-sensitive mechanistic hypothesis rather than a robust finding. Under the present model topology, CXCL10 is driven solely by IRF1 and does not receive STAT1 input. If STAT1-dependent CXCL10 induction and STAT1 → IRF1 feedback are included (see Section 4.7, Structural Sensitivity Analysis), the CXCL10 response to JAK inhibition reverses direction. The prediction that early JAK inhibition impairs viral control, however, is robust across both topologies.

Late JAK inhibition (S4) avoids viral rebound by allowing the early antiviral ISG response to mature before kinase blockade is introduced. However, its capacity to attenuate CXCL10 output in this framework is limited. While JAK inhibitors downregulate STAT1-dependent CXCL10 expression in vivo, our model emphasises that they leave the primary, RIG-I–IRF1-driven branch uninhibited. If high viral titres or lingering viral RNA continue to engage cytosolic sensors, upstream chemokine expression persists. Direct chemokine neutralisation via anti-CXCL10 monoclonal antibodies circumvents this signalling split entirely, dampening the pathogenic chemoattractant gradient regardless of which upstream transcriptional programme sustains it.

### 4.3. Combination Strategies: Antiviral Plus Immunomodulatory

The combination of IFN-*β* with anti-CXCL10 mAb (S7) achieves the most favourable profile among the eight simulated scenarios across all five outcome metrics. This strategy mirrors the combination approach used in COVID-19 (remdesivir plus baricitinib), although the mechanisms differ: the COVID-19 combination pairs a direct antiviral with an anti-inflammatory, whereas S7 pairs an immunostimulant with an anti-inflammatory. Complementarity between the antiviral and anti-inflammatory arms is imposed by the model structure and requires experimental testing. The practical implementation of S7 would require careful monitoring of CXCL10 levels to guide the timing and dose of the anti-CXCL10 component, as premature neutralisation before viral clearance could theoretically impair T-cell recruitment to the infected endothelium. No mathematical optimisation was performed. No objective function was defined, and no dose, administration-timing, or toxicity space was explored; the comparison is limited to the eight predefined scenarios of Table 2, and the model contains no adverse-effect variable, so stronger immune stimulation can appear beneficial provided the selected readouts improve.

The sequential RIG-I agonist → JAK inhibitor strategy (S8) is less effective than S7 because it fails to suppress CXCL10. This finding has a clear mechanistic explanation and a practical implication: if the goal is to reduce CXCL10 inflammation signals in hantavirus, the therapeutic target should be CXCL10 itself (or its receptor CXCR3), not the JAK–STAT pathway.

### 4.4. VCP/CB-5083: A Mechanism-Independent Alternative

The VCP inhibitor CB-5083 represents a qualitatively different therapeutic strategy: rather than modulating the host immune response, it targets a host-dependency factor required for viral replication [39]. This approach is in principle advantageous in immunocompromised patients, in whom IFN-based therapies may be less effective. Two caveats limit near-term translation: the Phase 1 oncology programme for CB-5083 was terminated for off-target effects on visual function, and VCP inhibition in vivo has been reported to delay viral clearance and to cause muscle toxicity in a lymphocytic choriomeningitis virus model. Host-dependency targeting therefore carries its own safety and timing trade-offs instead of escaping them. VCP is not modelled in the current ODE system because it acts upstream of the IFN-I cascade (at the level of viral assembly/egress), but its inclusion in a future extended model would be straightforward.

### 4.5. Limitations

Several limitations should be acknowledged. First, the transcriptomic data derive from a single cell type (HUVECs) and a single hantavirus species (HTNV); generalisation to other hantaviruses and cell types requires validation. Second, the ODE model is phenomenological: parameters are estimated from qualitative literature evidence rather than fitted to quantitative experimental data. Specifically, Equation (7) models CXCL10 production solely as a function of IRF1 activity to isolate primary pattern-recognition signalling. While biologically, CXCL10 transcription also receives substantial input from STAT1 via the IFNAR autocrine loop, omitting secondary STAT1-to-CXCL10 coupling allowed us to cleanly model the hazard of upstream PRR overactivation. Future multi-compartment models should incorporate dual IRF1/STAT1 promoter kinetics to evaluate partial kinase-mediated chemokine suppression. Equation (7) is not the only simplification of this kind: Equation (4) omits the STAT1 to IRF1 arm described in Section 3.2.3, even though IRF1 is itself an interferon-stimulated gene, and including it would lower IRF1 activity under JAK inhibition and could reverse the direction of the CXCL10 effect reported for S3. The model captures the topology and directionality of the IFN-I cascade but does not represent individual protein concentrations, post-translational modifications, or spatial compartmentalisation. Third, the model does not include pharmacokinetic/pharmacodynamic (PK/PD) components; drug onset is modelled as an instantaneous parameter switch instead of a concentration–time profile. Fourth, the drug–target interactions are based on known pharmacology and may not fully reflect hantavirus-specific biology. Fifth, the model does not represent adaptive immunity (T cells, B cells, antibodies), which plays a critical role in hantavirus disease outcome. Future work should incorporate PK/PD modelling, adaptive immune compartments, and parameter fitting to hantavirus-specific experimental data.

Sixth, the entire transcriptomic analysis is based on a single dataset using one cell type (HUVECs), one infection condition (72 h, MOI = 1), and one virus species (HTNV). Findings should not be automatically generalised to other orthohantaviruses (Puumala, Seoul, Andes, Sin Nombre) with different disease phenotypes and host responses. We performed internal robustness analyses (threshold sensitivity, leave-one-sample-out, STRING threshold sensitivity, and rank-based GSEA) to assess the stability of our conclusions within this dataset, but independent validation in alternative hantavirus datasets is needed. The scope of this study is accordingly restricted to Hantaan virus infection of endothelial cells and not to hantavirus infection generally.

### 4.6. Empirical Validation of Model Predictions

The what-if ODE model generates qualitative predictions about the relative efficacy of eight pharmacological interventions. Although the model parameters were not fitted to quantitative experimental data, the predictions can be validated against published in vitro and in vivo evidence from hantavirus and related viral systems. We assess each major prediction below and additionally evaluate the robustness of these predictions to parameter uncertainty through a systematic sensitivity analysis.

Early versus late IFN-β administration (S1 vs. S2). The model predicts that early IFN-β supplementation (S1, t = 0) reduces peak viral load by 27% (0.294 vs. 0.402) and accelerates clearance by 0.8 days (3.5 vs. 4.3 days), whereas late IFN-β (S2, t = 3 d) fails to reduce peak viral load (0.402) and provides only marginal improvement in clearance (4.0 vs. 4.3 days). This prediction is strongly supported by the work of Matthys and Mackow, who demonstrated that early IFN-β induction blocks hantavirus replication in human endothelial cells, whereas pathogenic hantaviruses (including HTNV) actively suppress early ISG induction, allowing the virus to establish replication before the IFN response is fully engaged [23]. Once viral replication is established, exogenous IFN has limited additional effect, as hantavirus-infected cells can become refractory to IFN treatment [45]. The model captures this temporal asymmetry through the logistic growth term: by day 3, viral load has already approached 80% of its peak, and the additional ISG induction from delayed IFN supplementation cannot reverse the established infection. Smee et al. similarly showed that pre-treatment with double-stranded RNA (a RIG-I agonist and IFN inducer) was more effective at reducing Hantaan virus titres than post-infection treatment [34], consistent with the model’s prediction that early intervention is critical.

JAK inhibitor timing and viral propagation (S3 vs. S4). The model predicts that early JAK inhibition (S3, t = 0) increases peak viral load by 17% (0.470 vs. 0.402) and delays clearance by 0.6 days (4.9 vs. 4.3 days), whereas late JAK inhibition (S4, t = 3 d) does not increase peak viral load (0.402) and causes only a modest delay in clearance (4.5 vs. 4.3 days). This prediction aligns with the findings of Ravlo et al., who demonstrated that JAK inhibitors can facilitate viral propagation by suppressing antiviral IFN signalling [11]. The timing-dependent effect is mechanistically explained by the model: during the first 3 days, endogenous IFN-β signalling through JAK1/JAK2 is critical for ISG induction and viral control. Blocking this axis early (S3) removes the primary antiviral defence, allowing unimpeded viral replication. By day 3, however, the IFN cascade has already induced substantial ISG expression, and the residual antiviral state persists even when JAK signalling is subsequently inhibited (S4). This prediction is consistent with the clinical experience with baricitinib in COVID-19, where the drug showed benefit when administered to hospitalised patients with established inflammation [44] but would be predicted to be harmful if administered during early viral replication—a concern shared by the hantavirus context.

RIG-I agonism reduces viral load (S5). The model predicts that enhancing RIG-I sensing (*k_R_* × 2.5, S5) reduces peak viral load by 23% (0.310 vs. 0.402) and accelerates clearance by 0.7 days (3.6 vs. 4.3 days). This prediction is directly supported by Kell et al., who showed that RIG-I-like receptor signalling drives the type I IFN response that limits HTNV replication [4]. Ma et al. further demonstrated that the long non-coding RNA NEAT1 enhances RIG-I signalling as a positive feedback loop during hantavirus infection [5], providing a host mechanism that the model’s RIG-I agonist scenario conceptually mimics. RIG-I agonists such as SB9200 (vesatolimod) and RGT100, which activate RIG-I directly, are currently in clinical development for chronic hepatitis B and would be testable in hantavirus models.

Anti-CXCL10 reduces CXCL10-associated inflammatory downstream pathways without affecting viral clearance (S6) and combination therapy (S7). The model predicts that increasing CXCL10 decay (*d_C_* × 5.0, S6) reduces peak CXCL10 by 73% (1.109 vs. 4.163) without altering peak viral load (0.402) or clearance time (4.3 days). This prediction is consistent with the clinical observation that CXCL10 is a marker of inflammatory activity rather than a direct antiviral effector. Jiang et al. reported that serum CXCL10 correlates with disease severity in hantavirus infection [35], and elevated CXCL10 is a hallmark of the capillary leak syndrome that characterises hantavirus cardiopulmonary syndrome (HCPS) and haemorrhagic fever with renal syndrome (HFRS). The model correctly captures the architectural separation between the antiviral axis (R → I → S → G → V) and the inflammatory axis (R → I → C): CXCL10 is a parallel output of RIG-I/IRF1 signalling that does not feed back on viral replication. The combination of early IFN-β supplementation with anti-CXCL10 (S7) achieves both reduced viral load (peak 0.294, clearance 3.5 days) and reduced CXCL10 (peak 0.671), representing the most favourable overall outcome among the eight simulated scenarios across both antiviral and anti-inflammatory metrics, suggesting that a dual strategy addressing both viral replication and CXCL10-associated inflammation may warrant investigation for severe hantavirus disease.

Sequential RIG-I agonist → JAK inhibitor therapy (S8). The model predicts that sequential administration of a RIG-I agonist (days 0–3) followed by a JAK inhibitor (from day 3) achieves viral control comparable to RIG-I agonist alone (peak 0.310, clearance 3.6 days) while reducing ISG AUC by 39% (90.6 vs. 148.9) and peak STAT1 by 42% (4.463 vs. 7.702) relative to S5. This prediction embodies the therapeutic concept of initiating antiviral signalling first to establish an ISG-mediated antiviral state, then dampening downstream inflammation once viral control is achieved. The model suggests that the ISG proteins induced during the first 3 days persist (with t½ ≈ 2.3 days) and continue to suppress viral replication even when new JAK/STAT signalling is inhibited, providing a mechanistic rationale for timed therapeutic sequencing.

### 4.7. Sensitivity Analysis and Robustness of Qualitative Predictions

To assess whether the qualitative predictions described above are robust to parameter uncertainty, we performed a systematic one-at-a-time sensitivity analysis. Each of the 15 model parameters was independently perturbed by factors of 0.5, 0.8, 1.2, and 1.5 (corresponding to −50%, −20%, +20%, and +50%), and all nine scenarios (baseline plus S1–S8) were re-evaluated for each perturbation, yielding 540 simulation runs plus the 9 nominal-parameter runs. We tracked 13 qualitative predictions spanning the key comparative relationships between scenarios (e.g., S1 clears faster than baseline, S3 increases peak viral load relative to baseline, S1 outperforms S2, S3 is worse than S4, S6 reduces CXCL10 without affecting clearance).

All 13 qualitative predictions held in 100% of the 61 parameter configurations tested (nominal plus 60 perturbations), indicating that the comparative rankings between scenarios are structurally robust to parameter uncertainty. The absolute values of individual metrics varied moderately: across all perturbations, the coefficient of variation (CV) for peak viral load ranged from 13.5% to 15.9%, for clearance time from 4.4% to 8.0%, for peak CXCL10 from 21.7% to 24.3%, and for ISG AUC from 22.5% to 23.6% across scenarios. These moderate CVs are expected given the ±50% perturbation magnitude, but the preservation of all qualitative rankings under such broad perturbation indicates that the model’s predictions arise from the architectural relationships between signalling modules and not from specific parameter values. This structural robustness is a desirable property for a *what-if* model intended to generate testable hypotheses more than precise quantitative predictions [12,13].

In addition to parameter sensitivity, we performed a structural sensitivity analysis to address the concern that the JAK/CXCL10 paradox (S3 increasing CXCL10) may depend on the model topology. An alternative topology was constructed by adding STAT1 → IRF1 feedback (*k_SI_* = 0.15) and STAT1 → CXCL10 induction (*k_SC_* = 0.10), reflecting the biological fact that IRF1 is itself an interferon-stimulated gene and that CXCL10 transcription receives STAT1 input. Under this alternative topology, every prediction concerning viral dynamics remained invariant, whereas several predictions concerning CXCL10 did not. The JAK/CXCL10 paradox was abolished instead of being reversed: early JAK inhibition (S3) left peak CXCL10 essentially unchanged relative to the alternative-topology baseline (7.03 vs. 7.17, a 2.0% decrease) instead of raising it by 27.7% as under the original topology, because JAK inhibition now also suppresses CXCL10 through the STAT1 → CXCL10 arm. The CXCL10 predictions for the interferon scenarios changed direction outright: measured against its own baseline, peak CXCL10 under early IFN-β (S1) rose by 463% under the alternative topology instead of falling by 40%, under late IFN-β (S2) rose by 683% instead of falling by 3%, and under the IFN-β plus anti-CXCL10 combination (S7) rose by 52% instead of falling by 84%. These reversals arise because adding STAT1 → IRF1 feedback creates a positive feedback loop that amplifies STAT1, and hence CXCL10, whenever STAT1 activation is pharmacologically boosted: peak STAT1 in S1 reaches 86.5 under the alternative topology against 8.45 under the original. By contrast, all predictions concerning peak viral load and clearance time were invariant across the two topologies, as were the CXCL10-reducing effect of anti-CXCL10 (S6: reductions of 73.4% and 76.8% under the original and alternative topologies, respectively) and the CXCL10-amplifying effect of RIG-I agonism (S5: increases of 63.9% and 63.5%). The antiviral conclusions of this study are therefore robust to this structural uncertainty, whereas every CXCL10 conclusion that depends on pharmacologically manipulating the interferon arm is topology-sensitive and should be read as a hypothesis generated under the present reduced topology rather than as a robust prediction (Supplementary Table S5, Figure S3).

### 4.8. Broader Methodological Contribution and Future Directions

Beyond the specific candidate therapies identified for Hantaan virus infection, this study illustrates a strategy for transforming heterogeneous biological knowledge into explicit mechanistic hypotheses that can subsequently be tested experimentally. The progressive workflow—transcriptomics to DEG selection to PPI network to hub prioritisation to druggability assessment to reduced mechanistic model to dynamic drug perturbation analysis—illustrates how accumulated biological and pharmacological knowledge can be organised into a model that supports testable therapeutic hypotheses. Even if individual components of this workflow have been used previously, their integration and application to hantavirus infection provides a meaningful research contribution.

The current network-reduction strategy could potentially be extended by incorporating additional secondary or aggregated biological knowledge sources. In the future, learned molecular representations such as protein language models may provide complementary information for identifying relationships not captured directly by transcriptomic differential expression or conventional PPI databases. Such extensions could further increase the utility of this analytical framework. The timing analysis presented here—comparing early versus delayed interventions across different pathway positions—illustrates a principal advantage of moving from static network analysis to dynamic simulation, and represents a direction that could be explored for other viral infections with similar IFN-centric host responses.

## 5. Conclusions

We present a three-stage computational framework for systematic drug repurposing in hantavirus infection. Network centrality analysis of the HTNV transcriptomic response identified 15 hub proteins in the IFN-I signalling cascade, of which 6 are druggable, yielding 12 repurposing candidates stratified by priority. ODE-based molecular what-if simulations of eight drug perturbation scenarios reveal that: (i) early IFN-*β* administration achieves the fastest viral clearance; (ii) under the assumption that CXCL10 is driven by IRF1 rather than by STAT1, early JAK inhibition is counterproductive, delaying clearance and elevating CXCL10; (iii) anti-CXCL10 monoclonal antibody selectively suppresses CXCL10 downstream signalling without affecting viral dynamics; and (iv) the combination of IFN-*β* with anti-CXCL10 mAb (Scenario 7) achieves the most favourable balance between antiviral efficacy and CXCL10 output among the eight simulated scenarios. Predictions (i) and (ii) concerning viral clearance are invariant to the structural sensitivity analysis of Section 4.7, whereas predictions (ii) and (iv) concerning CXCL10 are topology-sensitive and are advanced as hypotheses rather than as robust results. These findings provide a molecularly actionable prioritisation strategy for candidate therapies, which are offered as hypotheses for preclinical testing in Hantaan virus infection models.

## Figures and Tables

**Table 1:** Baseline ODE model parameters. All parameters are dimensionless or in units of day^−1^ unless otherwise noted.

Parameter	Symbol	Value	Interpretation
Viral replication rate	*r_V_*	1.2	Logistic growth rate
ISG antiviral suppression	*k*_MX1_	2.5	MX1-like viral clearance
RIG-I induction rate	*k_R_*	1.8	Viral RNA sensing
RIG-I half-activation	*K_R_*	0.3	Hill constant
Hill coefficient	*n*	2.0	Cooperative sensing
RIG-I decay	*d_R_*	0.4	Protein turnover
IRF1 activation rate	*k_I_*	1.5	RIG-I → IRF1
IRF1 decay	*d_I_*	0.5	Protein turnover
STAT1 phosphorylation	*k_S_*	1.2	IFN-*β* autocrine loop
JAK inhibitor effect	*k*_JAK_	0.0	0 = no drug
STAT1 dephosphorylation	*d_S_*	0.6	Phosphatase activity
ISG induction rate	*k_G_*	1.0	STAT1 → ISG
ISG decay	*d_G_*	0.3	mRNA/protein turnover
CXCL10 induction rate	*k_C_*	0.8	IRF1 → CXCL10
CXCL10 decay	*d_C_*	0.4	Secretion/clearance

**Table 2:** Drug perturbation scenarios for the molecular what-if analysis. Parameter changes are applied as multipliers to baseline values. *k*_JAK_ > 0 reduces STAT1 phosphorylation without affecting the IRF1–CXCL10 axis.

ID	Drug/Intervention	Mechanism	Parameter Change	Onset
S1	IFN-*α*/*β*	Boost STAT1 activation	*k_S_* × 3.0	*t* = 0
S2	IFN-*α*/*β*	Boost STAT1 activation	*k_S_* × 3.0	*t* = 3 d
S3	Baricitinib/Ruxolitinib	JAK1/2 inhibition	*k*_JAK_ = 0.8	*t* = 0
S4	Baricitinib/Ruxolitinib	JAK1/2 inhibition	*k*_JAK_ = 0.8	*t* = 3 d
S5	SB9200/RGT100 (RIG-I agonist)	Boost RIG-I sensing	*k_R_* × 2.5	*t* = 0
S6	Anti-CXCL10 mAb	CXCL10 neutralisation	*d_C_* × 5.0	*t* = 0
S7	IFN-*β* + anti-CXCL10 mAb	Dual: ISG boost + CXCL10 block	*k_S_* × 3.0; *d_C_* × 5.0	*t* = 0
S8	RIG-I agonist → JAK inhibitor	Sequential: boost then dampen	*k_R_* × 2.5 (*t* < 3 d); + *k*_JAK_ = 0.8 (*t* ≥ 3 d)	*t* = 0/3 d

**Table 3:** Top 15 hub proteins ranked by composite network centrality score. Degree: number of interaction partners in the STRING network. CNC score: composite min–max normalised score (Equation (1)). Druggability: Open Targets tractability score (0–4). Degrees, centralities, and ranks reported in this table and throughout Section 3.2 and Section 3.3 refer to the primary network of 176 nodes described in Section 2.2. Corresponding values for the networks rebuilt from the corrected gene sets are given in Supplementary Tables S2 and S3.

Gene	Rank	Degree	Betweenness	Closeness	CNC Score	Druggability
*ISG15*	1	100	0.0383	0.676	0.936	1
*IRF1*	2	92	0.0384	0.663	0.912	0
*CXCL10*	3	86	0.0394	0.639	0.902	4
*IFI35*	4	96	0.0340	0.663	0.866	0
*STAT1*	5	114	0.0229	0.726	0.791	2
*IFI44L*	6	67	0.0323	0.597	0.750	0
*MX1*	7	88	0.0246	0.651	0.722	2
*GBP1*	8	84	0.0242	0.632	0.703	2
*ZC3HAV1*	9	49	0.0312	0.543	0.674	0
*TRIM25*	10	51	0.0292	0.550	0.657	0
*DDX58*	11	99	0.0162	0.676	0.652	2
*PSMB9*	12	64	0.0242	0.581	0.636	0
*BATF2*	13	36	0.0303	0.515	0.621	0
*EIF2AK2*	14	76	0.0192	0.616	0.613	0
*BST2*	15	70	0.0199	0.601	0.603	0

**Table 4:** Drug repurposing candidates for hantavirus infection. Drugs are grouped by priority tier. Approval: regulatory status as of 2026.

Drug	Class	Mechanism	Impacted Network Proteins	Approval	Tier
Ribavirin	Nucleoside analogue	Viral RNA synthesis inhibitor	OAS1, MX1	FDA approved	1
Remdesivir	Nucleoside analogue	RNA polymerase inhibitor	DDX58, IRF1	FDA approved	1
Favipiravir	Pyrazine carboxamide	RNA polymerase inhibitor	DDX58, IRF1	Approved (some countries)	1
Interferon-*α*	Protein therapeutic	Type I IFN receptor agonist	ISG15, IRF1, STAT1, MX1, OAS1, IFIT1	FDA approved	1
Interferon-*β*	Protein therapeutic	Type I IFN receptor agonist	ISG15, IRF1, STAT1, MX1, OAS1, IFIT1	FDA approved	1
Baricitinib	Small molecule	JAK1/JAK2 inhibitor	STAT1	FDA approved	2
Ruxolitinib	Small molecule	JAK1/JAK2 inhibitor	STAT1	FDA approved	2
Tofacitinib	Small molecule	JAK1/JAK3 inhibitor	STAT1	FDA approved	2
CB-5083	Small molecule	VCP/p97 inhibitor	VCP	Phase 1, terminated	3
SB9200	Small molecule	RIG-I agonist	DDX58	Discontinued (Phase 2)	4
RGT100	Small molecule	RIG-I agonist	DDX58	Phase 1	4
Anti-CXCL10 mAb	Monoclonal antibody	CXCL10 neutralisation	CXCL10	Preclinical/Phase 1	4

**Table 5:** Summary metrics for the eight molecular what-if scenarios. Peak viral load: maximum of *V*(*t*) over 14 days. Clearance: first time at which *V* < 0.01. Peak CXCL10: maximum of *C*(*t*). ISG AUC: $\int_{0}^{14}G\left(t\right)\;\mathrm{dt}$. Peak STAT1: maximum of *S*(*t*). Baseline values shown in the first row. The direction of desirability is defined biologically as follows: lower peak viral load, shorter clearance time and lower peak CXCL10 are treated as favourable. ISG AUC and peak STAT1 have no intrinsic direction of desirability, since higher values denote stronger antiviral signalling but also greater potential for inflammatory injury; emphasis in those two columns therefore marks the extreme value and not the preferred one.

Scenario	Peak Viral	Clearance	Peak CXCL10	ISG AUC	Peak STAT1
	Load	(Days)	(Norm.)		(Norm.)
Baseline (no drug)	0.402	4.3	4.163	88.8	4.689
S1: IFN-*α*/*β* (early, *t* = 0)	0.294	3.5	2.495	164.0	8.454
S2: IFN-*α*/*β* (late, *t* = 3d)	0.402	4.0	4.033	243.0	13.381
S3: JAK inhibitor (early, *t* = 0)	0.470	4.9	5.315	62.0	3.319
S4: JAK inhibitor (late, *t* = 3d)	0.402	4.5	4.217	53.3	2.696
S5: RIG-I agonist (SB9200/RGT100)	0.310	3.6	6.822	148.9	7.702
S6: Anti-CXCL10 mAb	0.402	4.3	1.109	88.8	4.689
S7: IFN-*β* + anti-CXCL10 (combo)	0.294	3.5	0.671	164.0	8.454
S8: RIG-I agonist → JAK inh. (seq.)	0.310	3.6	6.825	90.6	4.463

## Data Availability

The transcriptomic data used in this study are publicly available from the Gene Expression Omnibus (GEO accession GSE133751). The analysis code for DESeq2 processing, STRING network construction, centrality calculations, Open Targets extraction, ODE integration, sensitivity analyses, and figure generation is provided as supplementary scripts. Software versions: DESeq2 1.46.0 (R 4.4.1), Python 3.11, SciPy 1.14, STRING-db v12.0, Open Targets Platform v24.x (accessed 30 November 2025). The complete DESeq2 output for all 16,510 genes tested is provided in Supplementary Table S1, enabling readers to reproduce alternative thresholding and rank-based enrichment analyses.

## Supplementary Materials

The following supporting information can be downloaded at: https://www.mdpi.com/article/10.3390/v18101100/s1, Supplementary Table S1: Complete DESeq2 output for GSE133751 (HTNV vs. mock HUVECs; *n* = 3 per group), for all 16,510 genes tested. FDR-significant genes: padj < 0.05 and |log_2_FC| ≥ 1 (*n* = 358). Nominally significant gene set used to seed the primary network: *p* < 0.05 and |log_2_FC| ≥ 1 in the initial RPKM-based analysis (*n* = 203). Supplementary Table S2: STRING network sensitivity analysis at combined confidence thresholds 0.4 (296 nodes) and 0.7 (216 nodes), with degree, betweenness, closeness, and composite network centrality scores for every node. Supplementary Table S3: Hub-rank stability analysis across four DEG-inclusion thresholds and leave-one-sample-out resampling. Supplementary Table S4: Rank-based gene set enrichment analysis (fgsea) over the full log_2_ fold-change distribution. Supplementary Table S5: Structural sensitivity analysis comparing the original and alternative ODE topologies across all five outcome metrics and all nine scenarios. Figure S1: Principal component analysis of HTNV-infected versus mock HUVECs. Figure S2: GSEA enrichment plot for the type I interferon response. Figure S3: Structural sensitivity comparison bar plots.

## Abbreviations

The following abbreviations are used in this manuscript:

CNC	Composite network centrality
DEG	Differentially expressed gene
GEO	Gene Expression Omnibus
GO:BP	Gene Ontology Biological Process
HCPS	Hantavirus cardiopulmonary syndrome
HFRS	Haemorrhagic fever with renal syndrome
HTNV	Hantaan virus
HUVEC	Human umbilical vein endothelial cell
IFN-I	Type I interferon
ISG	Interferon-stimulated gene
mAb	Monoclonal antibody
ODE	Ordinary differential equation
PPI	Protein–protein interaction
RdRp	RNA-dependent RNA polymerase
RIG-I	Retinoic acid-inducible gene I
VCP	Valosin-containing protein
